# The impact of maternal disgust sensitivity from pregnancy until 3 years postpartum on the early development of disgust sensitivity in the child

**DOI:** 10.3389/fpsyg.2025.1680260

**Published:** 2025-11-27

**Authors:** Šárka Kaňková, Ashkan Latifi, Daniela Dlouhá, Jana Benešová, Jana Ullmann, Pavel Calda

**Affiliations:** 1 Department of Philosophy and History of Science, Faculty of Science, Charles University, Prague, Czechia; 2 Department of Gynaecology, Obstetrics and Neonatology, First Faculty of Medicine, Charles University and General University Hospital, Prague, Czechia

**Keywords:** disgust sensitivity in children, emotion, pathogens, parental modeling, parity, siblings, stress

## Abstract

**Introduction:**

Disgust plays a key role in pathogen avoidance. In children, it starts to develop alongside cognition, emotion processing, and social skills around the third year. This study examined whether maternal disgust sensitivity predicts disgust sensitivity in 3-year-old children and whether this relationship is affected by parity and maternal psychological factors.

**Methods:**

Data were collected from 163 women (60% primiparae) who repeatedly (in the first trimester of pregnancy and then 6 weeks, 1 year, and 3 years postpartum) completed questionnaires on disgust sensitivity, state anxiety, positive and negative affectivity, and perceived stress. At 3 years postpartum, mothers also completed the Child Disgust Scale.

**Results:**

Path models showed that maternal disgust sensitivity at each time point significantly predicted maternal disgust sensitivity at the next point, and maternal disgust sensitivity 3 years postpartum significantly predicted children's disgust sensitivity. Higher disgust was also reported in children of multiparous mothers. Moreover, perceived stress and negative affectivity in the first trimester positively predicted first-trimester maternal disgust sensitivity.

**Discussion:**

Our results suggest that children's disgust sensitivity may be shaped by maternal disgust sensitivity and maternal psychological factors in early pregnancy may play a secondary role in this process. Older siblings may also play a role by serving as models.

## 1 Introduction

Disgust is an emotion that plays a central role in shaping how individuals respond to environmental threats, particularly those associated with contamination and disease. While traditionally viewed through the evolutionary lens as an adaptation for disease avoidance (Curtis et al., 2011), disgust is increasingly recognised as an emotional trait that emerges early in development and is shaped by both biological predispositions and environmental influences (Oaten et al., 2009). Like other biological traits, disgust sensitivity, especially its state component reflected in response intensity, varies between individuals and can change over the course of life. Individuals with higher levels of trait disgust tend to exhibit stronger emotional reactions when exposed to disgust inducing stimuli, which indicates a connection between dispositional sensitivity and momentary emotional responses (Stark et al., 2005). The main factors that can influence disgust sensitivity include age (Quigley et al., 1997; Polák et al., 2019), sex (Al-Shawaf et al., 2018), health status (Stevenson et al., 2009; Dlouhá et al., 2023), immunity (Kanková et al., 2022), hormones (Kanková et al., 2023a, 2024), reproductive status (Dlouhá et al., 2024), and pathogen threat (Cepon-Robins et al., 2021; Kanková et al., 2023b).

In young children, disgust sensitivity seems to develop in tandem with cognitive, emotional, and social maturation (Sawchuk, 2009). After birth, the newborn's immune system is not yet fully developed. Infants are protected by substances present in the breast milk but also by the mother's behavioural immune system including heightened maternal disgust, which may help shield the infant from pathogens (Al-Shawaf et al., 2018). A stronger pressure aiding the development of disgust emerges as children gradually gain independence, which correlates with their cognitive development and growing ability to understand the causes and consequences of potential infections. Researchers found that children around the age of three expressed disgust in response to stimuli related to food, hygiene violations, and body secretions (Stevenson et al., 2010). By the age of four, disgust responses extended to animal-related stimuli (worms and body envelope violations), while sociomoral stimuli began to elicit disgust around the age of seven.

Of particular interest is the potential role of maternal factors in influencing the development of disgust sensitivity in early childhood because children's disgust responses may be shaped not only by inherited genetic predispositions but also by how their caregivers express, model, and reinforce disgust-related behaviours (Askew et al., 2014; Sherlock et al., 2016; Tybur et al., 2020). Through repeated exposure to caregiver's emotional reactions and behavioural cues, especially in early childhood, when parents play a central role in socialisation, children are likely to adopt similar responses. Disgust expressions of the adults are often visible and salient, making them easily noticed and imitated by young children. A study showed that parents of younger children tended to display stronger disgust reactions, which suggests a training aspect of this behaviour (Stevenson et al., 2010). Similarly, parents of children aged 2 to 3 years expressed exaggerated avoidance and overt disgust marked by intense facial expressions, vocalisation, and gestures, when exposed to disgust-eliciting stimuli (Oaten et al., 2014). Overall, children come to understand and experience disgust and all its aspects gradually—and parents play a key role in the process. This is further supported by the findings of Alladin and colleagues (Alladin et al., 2024) which suggest that behavioural avoidance of disgusting stimuli takes place before any physiological reaction of disgust, which indicates that children's early reactions are shaped by observing and imitating parental cues. Over time, they may evolve into internalised, bodily feelings of disgust. Still, these results should be interpreted with caution: the authors admit that they may have used stimuli that were strong enough to trigger visual avoidance but not intense enough to elicit a measurable gastrointestinal response.

Aside from serving as a direct model, maternal psychological traits may shape disgust sensitivity in mothers and children also indirectly. The role of parents in children's emotional development is well documented (Oaten et al., 2014; Reschke et al., 2024) and especially maternal mental health—including conditions such as anxiety, stress, and negative emotional states such as depression—has been linked to children's emotional regulation and broader socio-emotional characteristics (Stack et al., 2010; Hajal and Paley, 2020; Yakupova and Suarez, 2023). Longitudinal studies have shown that mother's psychological wellbeing and emotional disposition during pregnancy and the postnatal period can influence various outcomes in her child, including temperament, internalising behaviours, and social-emotional functioning (Glynn et al., 2018; Rees et al., 2019; Rusanen et al., 2024). Various psychological constructs, such as perceived stress, state anxiety, and negative affect, are known to be associated with increased emotional reactivity and negative moods (van Eck et al., 1998; Xie et al., 2024) but also reduced cognitive control (Luethi, 2008; Zhu et al., 2022). Both anxiety and disgust sensitivity elicit avoidance behaviours, often in response to overlapping or similar types of stimuli. Several studies found that higher disgust sensitivity correlates positively with anxiety-related disorders (Olatunji et al., 2010; Davey et al., 2008; Olatunji et al., 2007a). Given that both anxiety and disgust sensitivity involve avoidance-based responses to perceived threats, it is plausible that children may adopt similar sensitivities through observational learning or emotional alignment with their mother's distress. Although this relationship has not been directly studied, evidence suggests that heightened maternal stress or anxiety may indirectly contribute to increased disgust sensitivity in children, potentially through emotional modelling, shared affective environments, or disrupted emotion regulation pathways.

Another factor of relevance is parity; it has been associated with differences in caregiving practices, risk perception, and maternal responsiveness (Maupin et al., 2016). Some research suggests that first-time mothers exhibit higher levels of caution, anxiety, and emotional vigilance but also lower levels of control and self-efficacy than multiparous women (Fish and Stifter, 1993; Shakarami et al., 2021; Green et al., 2022; Lindblad et al., 2022), which may lead to more modelling of avoidant or disgust-based behaviours. Nevertheless, the results of studies that investigated the relationship between parity and maternal disgust sensitivity are inconsistent: some studies conducted on pregnant women (Dlouhá et al., 2023; Kanková et al., 2023b) indicate that multiparous women have a higher disgust sensitivity than primiparous women because their disgust is elevated in order to protect their other offspring. On the other hand, other studies conducted on the general population found that childless people report higher levels of disgust than parents (Stefanczyk et al., 2024; Prokop and Fančovičová, 2016). Stefanczyk and colleagues suggest that increased disgust sensitivity could be specific to pregnancy, while later contact with disgust-inducing stimuli (such as diaper changing) may lead to the opposite habituation process.

Despite this growing interest in disgust development, only few studies so far examined how maternal disgust sensitivity during pregnancy and other maternal psychological aspects relate to the disgust sensitivity of the child. To address this gap, the present study adopted a longitudinal design to assess how maternal disgust sensitivity and associated psychological traits (measured at multiple time points—from pregnancy through 3 years postpartum) predict disgust sensitivity of 3-year-old children. Specifically, we investigated whether maternal disgust sensitivity is a direct predictor of children's disgust sensitivity. Additionally, we looked at how parity and affective states (maternal anxiety, perceived stress, and positive and negative affect) influence disgust sensitivity in both the mother and the child. Based on previous studies, we hypothesise that there will be a positive correlation between disgust sensitivity in mothers and their children and that parity and maternal affective states will influence this relationship.

## 2 Materials and methods

### 2.1 Participants and procedures

Participants were recruited in their first trimester of pregnancy in collaboration with private gynaecological clinics in Prague between June 2019 and June 2021 (Sample 1, S1) and in the Center of Fetal Medicine and Ultrasound Gynecological in the General University Hospital in Prague between March 2020 and January 2021 (Sample 2, S2). They were initially recruited for a larger study focused on pregnant women. Only women aged 18–45 years with singleton pregnancies and without serious health problems were included in the study.

All women were informed about the aims of the study and provided written informed consent with participation. They were also ensured that they could withdraw from the study at will at any point. Because the study included a postpartum follow-up (the main phase of data collection for this study), participants also agreed to be contacted again to complete additional questionnaires. The women completed a questionnaire during the first trimester of pregnancy and then follow-up questionnaires at 6 weeks postpartum, 1 year postpartum, and 3 years postpartum. They were compensated 200 CZK (9 USD) for completing the online questionnaire at 3 years postpartum. The study was approved by the Institutional Review Board of the Faculty of Science, Charles University (no. 2022/04) and by the Ethics Committee of General University Hospital in Prague (No. 384/16; 92/17).

**Figure 1:**
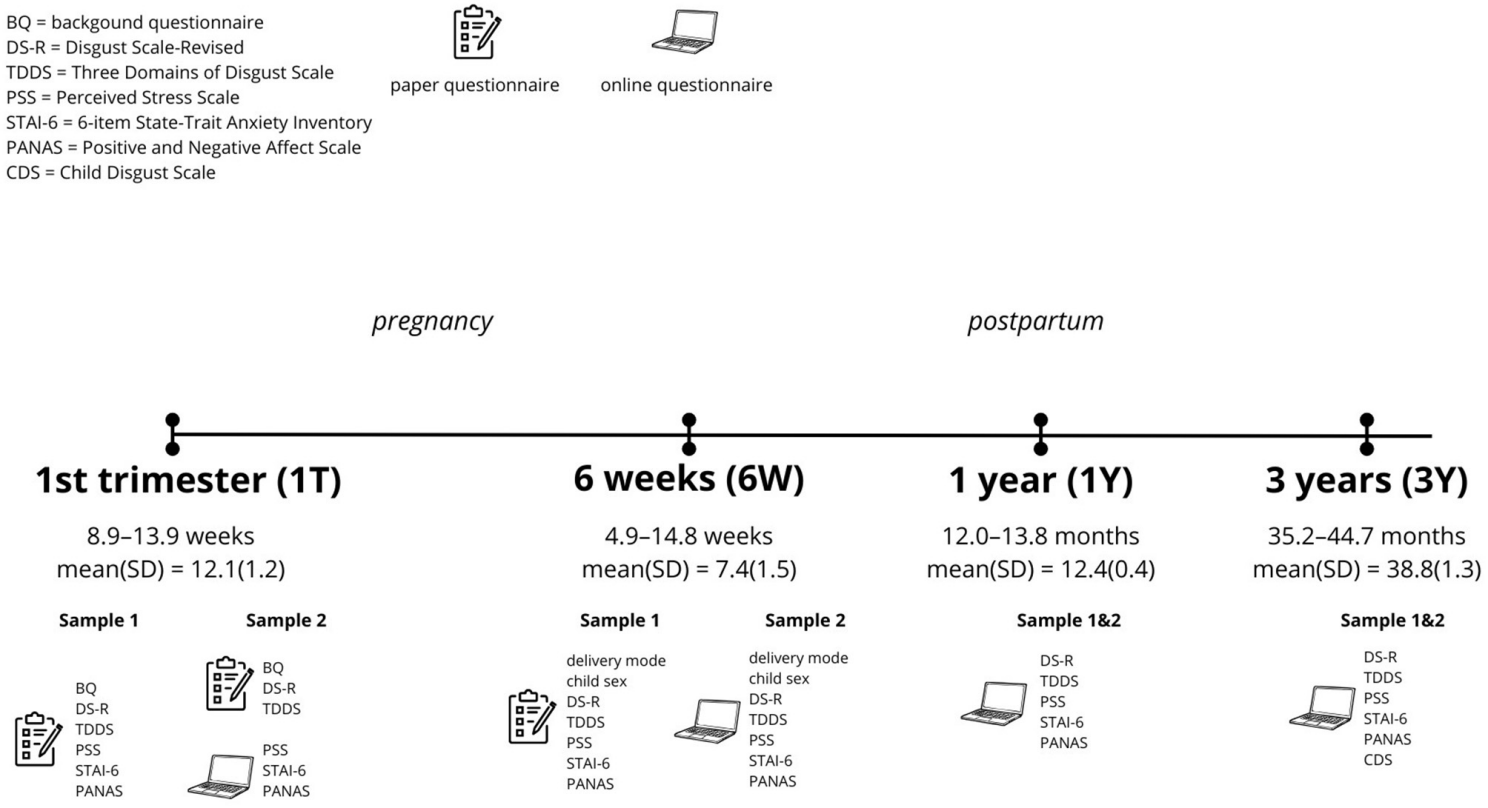
Graphical timeline of the study.

Women completed the first questionnaire in paper form (women in S2 completed both a paper questionnaire and a separate online survey, which included the measures of state anxiety, stress, and positive and negative affectivity) in the first trimester of pregnancy (1T: 8.9–13.9 weeks, mean = 12.1, SD = 1.2). The second questionnaire was completed 6 weeks postpartum (6W: 4.9–14.8 weeks, mean = 7.4, SD = 1.5). Participants in S1 completed the second questionnaire in paper form during their in-person visit to the gynaecology outpatient clinic, while participants in S2, who were initially recruited at the Center of Fetal Medicine, completed it online as they subsequently attended different gynaecological facilities and were included in the follow-up study only online. The third questionnaire was completed 1 year postpartum in an online form (1Y: 12.0–13.8 months, mean = 12.4, SD = 0.4). The fourth questionnaire was completed 3 years postpartum, also online (3Y: 35.2–44.7 months, mean = 38.8, SD = 1.3). At each timepoint, the women completed questionnaires measuring levels of disgust sensitivity, state anxiety, perceived stress, and positive and negative affectivity. At 3 years postpartum, they also completed a questionnaire on disgust sensitivity of their child. The study timeline, summarizing all data collection phases and the timing of administered questionnaires, is shown in Figure 1. The individual standardised questionnaires are described in the Questionnaires section.

A total of 163 women in S1 and 398 women in S2 were initially recruited as part of another study focused on pregnancy. Of these, 79 women (S1) and 274 women (S2) agreed to be contacted in the future to complete additional questionnaires. Ultimately, 42 women (S1) and 121 women (S2) completed the questionnaire at 3 years postpartum, which served as the inclusion criterion for this study. For final sample characteristics (age, pre-pregnancy weight, parity, child sex, delivery mode, education level, and monthly household income), see Table S1. Women were 21–42 years old (mean = 32.2, SD = 4.1) at the time of recruitment, 77.8% had a university degree, 59.5% were primiparas, 53.7% gave birth to a girl, and 65.6% had a spontaneous vaginal birth. In our sample, no children had congenital or serious health problems that could have influenced the study results. Mothers who completed the 3-year follow-up questionnaire also reported on their child's health and development, and none of the preterm children or those with low birth-weight (*N* = 8) were described as having conditions warranting exclusion from the study.

### 2.2 Questionnaires

#### 2.2.1 General anamnestic and sociodemographic questionnaire

A paper-based background questionnaire was administered to the women at recruitment, collecting general sociodemographic (age, education, income) and other background (weight, parity) information. Additional information regarding delivery mode and child sex was obtained from the questionnaire 6 weeks postpartum.

#### 2.2.2 Child disgust scale—maternal report

To assess the children's disgust sensitivity, a modified version of the Child Disgust Scale (CDS) (Viar-Paxton et al., 2015) was used in 3Y. The scale contains 14 items—descriptions of different situations and subsequent behaviours. Out of those situations, nine are linked to disgust avoidance (“If a dog licked my popsicle I would still eat it”) and five are linked to disgust affect (“When I see blood I feel dizzy”), creating two subscales. The children are asked to rate how often they would do what the description says on a three-point scale; Always (0), Sometimes (1), Never (2). Five items are reverse scored. The scores are summed and can range from 0 to 28 for the Total score, 0 to 18 for Disgust Avoidance and 0 to 10 for Disgust Affect. Higher scores indicate higher disgust sensitivity. For the purposes of this study, the scale was translated to Czech by two independent bilingual translators and was modified for maternal report—the participants were asked how they think their child would behave in the stated situations. They were explicitly asked to answer how their child would really behave and not how they would want them to behave.

#### 2.2.3 Disgust scale-revised

As one of the measures of adult disgust sensitivity, we used the Disgust Scale-Revised (DS-R) (Olatunji et al., 2007b), specifically the Czech language version (Polák et al., 2019). This scale includes 25 disgust-related items, which are scored by the participants on a 5-point (0–4) Likert scale, rating either how much they agree with given statements (i.e. “It bothers me to hear someone clear a throat full of mucous”; 0 = Strongly disagree, 4 = Strongly agree) or how disgusting they find a described situation (i.e. “You see maggots on a piece of meat in an outdoor garbage pail”; 0 = Not disgusting at all, 4 = Extremely disgusting). These items can be categorised into three separate subscales: the Core subscale (12 items, score ranging from 0 to 48), the Animal Reminder subscale (8 items, score ranging from 0 to 32) and the Contamination subscale (5 items, score ranging from 0 to 20). The total score can range from 0 to 100, with higher score indicating higher disgust sensitivity.

#### 2.2.4 Three domains of disgust scale

Another questionnaire we used to measure disgust sensitivity in adults is the 21-item Three Domains of Disgust Scale (TDDS) (Tybur et al., 2009). This questionnaire focuses on the Pathogen, Sexual and Moral domains of disgust. For each domain, there are 7 items. Each item is a statement that is rated on a 7-point Likert scale, ranging from 0 (“Not disgusting at all”) to 6 (“Extremely disgusting”). The score of each domain can range from 0 to 42, with higher score indicating higher disgust sensitivity. The scale was translated to Czech by two independent bilingual translators.

#### 2.2.5 State trait anxiety inventory

To assess state anxiety, the 6-item scale derived from the State-Trait Anxiety Inventory (STAI) (Spielberger et al., 1983); the STAI-6 (Marteau and Bekker, 1992) was used. The STAI-6 contains six items, describing how someone could feel at a given moment and participants rate, on a 1–4 scale, how true these statements are about them (Not at all, Somewhat, Moderately, Very much). Three items are reverse scored. The score is a sum of all item responses and can range from 6-24, with higher scores indicating higher state anxiety. The original scale was translated into Czech by two independent bilingual translators, we have verified its accuracy by backtranslation and successfully used it in previous research (Takács et al., 2024, 2025a,b).

#### 2.2.6 Perceived stress scale

The Perceived Stress Scale (PSS) (Cohen et al., 1983) questionnaire was used to measure levels of perceived stress. This questionnaire contains 10 items, which inquire about how often the participant found situations in their life to be unexpected, uncontrollable or stressful (i.e. “How often have you been upset because of something that happened unexpectedly?”). In the original questionnaire, these items relate to the last month, but for the purposes of this study, the participants were asked about the past 2 weeks. Each item is rated on a 5-point Likert scale, ranging from 0 to 4 (Never, Almost never, Sometimes, Fairly often, Very often). Four items are reverse scored. The total score can range from 0 to 40, with higher score indicating higher intensity of perceived stress. The original scale was translated into Czech by two independent bilingual translators, we have verified its accuracy by backtranslation and successfully used it in previous research (Takács et al., 2024, 2025b).

#### 2.2.7 Positive and negative affect scale

To measure the two primary dimensions of mood—positive and negative affect, the Positive and Negative Affect Scale (PANAS) (Watson et al., 1988) was used. The scale has 20 items, describing different feelings or emotions, with 10 relating to Positive Affect (PA) and 10 to Negative Affect (NA). The participants are asked how often they experienced these feelings in the past week, which they rate on a 5-point Likert scale, ranging from 1 to 5 (Very slightly or not at all, A little, Moderately, Quite a bit, Very much). The scores are calculated as a sum for each affect scale and can range from 10 to 50, with higher scores indicating higher intensity of that particular affect. The original scale was translated into Czech by two independent bilingual translators, we have verified its accuracy by backtranslation and successfully used it in previous research (Takács et al., 2024, 2025b).

### 2.3 Statistical methods

All statistical analyses were conducted using non-parametric and model-based approaches in line with the distributional properties of the dependent variables, and via R Version 4.4.2.

Across all questionnaires used in the study, missing data were addressed using a consistent approach. If less than 20% of items were missing within a subscale/domain or overall score, the mean of the completed items in that subscale/domain or overall score was imputed for the missing responses (the maximum number of imputed items was 14, in the case of the CDS). If more than 20% of items were missing, the corresponding score was excluded from the analysis (the maximum number of excluded participants was 11, also in the case of the CDS).

**Figure 2:**
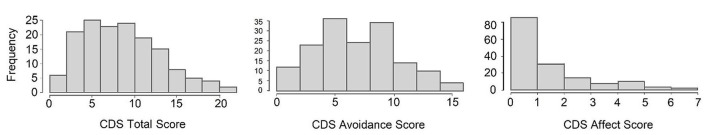
Histograms of total, avoidance, and affect CDS scores.

Preliminary data exploration revealed that the main outcome variables—Total CDS, CDS Avoidance subscale, and CDS Affect subscale—did not follow a Gaussian distribution, as assessed visually via histograms (Figure 2). Consequently, for comparable results across all variables, non-parametric Kendall's correlation or a non-parametric Mann-Whitney U test analyses were conducted to assess the effects of demographic variables such as mother's age at 3 years postpartum, nor education, child's sex, delivery mode, or the place of recruitment (i.e., differences between samples S1 and S2). Path models were estimated to test hypothesised mediational and longitudinal relationships between maternal, demographic, and child variables. Path modelling is a statistical technique within the broader family of structural equation modelling (SEM) that allows the simultaneous estimation of multiple direct and indirect relationships among observed variables. This approach enables the decomposition of effects (e.g., mediating pathways) and the assessment of longitudinal continuity across repeated measures.

Given the large number of correlation analyses, corrections for multiple testing were systematically applied to control for false positives. The Benjamini-Hochberg procedure was used to adjust *p*-values, with the false discovery rate (FDR) set at 0.1. This method is designed to limit the expected proportion of false discoveries (Type I errors) among the rejected hypotheses, providing a balance between statistical power and error control when conducting multiple comparisons. The dataset presented in this study is available in the online repository Figshare at 10.6084/m9.figshare.29590139.

## 3 Results

### 3.1 Descriptive statistics

At 3Y, the mean total CDS score was 9.01 (SD = 4.66), with subscale means 1.62 (SD = 1.72) for Affect and 7.31 (SD = 3.53) for Avoidance. Internal consistency was acceptable to good in all subscales (Cronbach's α = 0.68 to 0.80), indicating a reliable measurement. For further details, see Table S2.

Measures related to maternal disgust sensitivity (DS-R and TDDS scores) showed an adequate internal consistency at all timepoints, with Cronbach's α values mostly above 0.6, indicating an acceptable reliability. Mean scores for the DS-R subscales and the Total DS-R score remained relatively stable from 1T to 3Y, with means ranging from 49.05 to 51.81. Mean scores of the TDDS varied slightly over time, with a tendency for higher scores in Moral and Pathogen domains at later timepoints. Descriptive statistics for all measured maternal disgust scores at all time points and individual Cronbach's α values are presented in Table S3.

Maternal affective states, including STAI, PSS, PA and NA showed good internal consistency (Cronbach's α = 0.84 to 0.90) at different timepoints. STAI scores were relatively stable, with mean scores around 10 to 11 at all timepoints, while PSS scores showed an increasing trend from pregnancy (M = 12.89) to 3 years postpartum (M = 17.30), suggesting that perceived stress rises during the early parenting period. NA scores were generally lower in the earlier stages and peaked slightly at 3Y (M = 19.80), while PA scores were higher and more stable throughout. Descriptive statistics for all measured maternal affective states at all timepoints and Cronbach's α values are presented in Table S4.

### 3.2 Associations between demographics and disgust sensitivity in children

The results indicated that parity was significantly and positively associated with CDS Total scores (Mann-Whitney *U* = 2208, *p* = 0.043) and with Affect subscale (Mann-Whitney *U* = 2233, *p* = 0.032). Multiparity was associated with higher reported levels of children's disgust sensitivity.

No other demographic variables were significantly associated with CDS scores: neither mother's age at 3 years postpartum, nor education, child's sex, delivery mode, or the place of recruitment (i.e. differences between samples S1 and S2) showed any significant associations with any of the disgust subscales (*p* > 0.05).

After applying the Benjamini-Hochberg correction for multiple comparisons, no significant results remained statistically significant (see Table S5).

### 3.3 Correlation between maternal disgust and disgust sensitivity in children

Significant positive correlations were observed between maternal disgust sensitivity measured using DS-R and children's disgust sensitivity at multiple timepoints, whereby the strongest and most consistent associations with CDS scores were observed in maternal disgust sensitivity scores at 3Y. Total DS-R at this timepoint significantly correlated with both CDS subscales (Affect: τ = 0.347, *p* < 0.001; Avoidance: τ = 0.216, *p* < 0.001) and the Total CDS score (τ = 0.281, *p* < 0.001). All subscales of the DS-R (Core, Contamination, and Animal Reminder) were likewise significantly correlated with all CDS scores (*p* < 0.01 to *p* < 0.001) at 3Y. The results of all correlations, including all DS-R scores at all measured timepoints, are shown in Table 1.

**Table 1:** Correlations between maternal (DS-R, TDDS) and children's (CDS) disgust sensitivity.

**Variables**	**Total CDS**		**Affect CDS**		**Avoidance CDS**	
	**Tau**	***p***	**Tau**	***p***	**Tau**	***p***
1T total DS-R	0.186	0.001	0.220	0.000	0.141	0.010
1T core DS-R	0.100	0.070	0.175	0.002	0.072	0.185
1T contamination DS-R	0.258	0.000	0.156	0.005	0.247	0.000
1T animal reminder DS-R	0.153	0.006	0.156	0.005	0.121	0.026
1T pathogen TDDS	0.093	0.091	0.093	0.090	0.077	0.156
1T sexual TDDS	0.132	0.016	0.007	0.901	0.170	0.002
1T moral TDDS	0.090	0.103	0.131	0.017	0.072	0.185
6W total DS-R	0.138	0.052	0.162	0.022	0.132	0.062
6W core DS-R	0.080	0.260	0.185	0.009	0.054	0.446
6W contamination DS-R	0.196	0.006	0.088	0.212	0.220	0.002
6W animal reminder DS-R	0.116	0.103	0.114	0.107	0.105	0.140
6W pathogen TDDS	0.088	0.221	0.132	0.064	0.076	0.286
6W sexual TDDS	0.153	0.033	0.178	0.012	0.117	0.101
6W moral TDDS	0.069	0.334	0.018	0.804	0.123	0.084
1Y total DS-R	0.207	0.006	0.237	0.002	0.163	0.031
1Y core DS-R	0.149	0.049	0.254	0.001	0.102	0.173
1Y contamination DS-R	0.175	0.021	0.125	0.098	0.166	0.027
1Y animal reminder DS-R	0.157	0.039	0.145	0.056	0.119	0.113
1Y pathogen TDDS	0.113	0.135	0.150	0.048	0.082	0.275
1Y sexual TDDS	0.212	0.005	0.209	0.006	0.173	0.022
1Y moral TDDS	0.000	0.997	−0.002	0.981	0.036	0.628
3Y total DS-R	0.281	0.000	0.347	0.000	0.216	0.000
3Y core DS-R	0.221	0.000	0.308	0.000	0.158	0.003
3Y contamination DS-R	0.279	0.000	0.250	0.000	0.258	0.000
3Y animal reminder DS-R	0.226	0.000	0.267	0.000	0.167	0.002
3Y pathogen TDDS	0.161	0.003	0.171	0.002	0.149	0.006
3Y sexual TDDS	0.214	0.000	0.183	0.001	0.202	0.000
3Y moral TDDS	0.076	0.166	0.005	0.921	0.104	0.055

The abbreviations refer to various stages of pregnancy and postpartum: 1T, first trimester of pregnancy; 6W, 6 weeks postpartum; 1Y, 1 year postpartum; 3Y, 3 years postpartum. Significant correlations are bolded. Those correlations which remained significant after applying Benjamini-Hochberg correction for multiple testing are underlined, FDR was set at 0.1.

Regarding correlations between maternal disgust sensitivity measured by the TDDS and the CDS, the Pathogen domain at 3Y was significantly positively associated with all CDS scores (Total CDS: τ = 0.161, *p* = 0.003; Affect: τ = 0.171, *p* = 0.002; Avoidance: τ = 0.149, *p* = 0.006) but Pathogen domain at 1Y was significantly positively associated only with the CDS Affect subscale (τ = 0.150, *p* = 0.048). Significant positive correlations were also observed between the Sexual domain and CDS at multiple timepoints, with the strongest correlation observed at 3Y (Total CDS: τ = 0.214, *p* < 0.001; Affect: τ = 0.183, *p* = 0.001; Avoidance: τ = 0.202, *p* < 0.001). The results of all correlations, including all TDDS scores, are shown in Table 1.

Importantly, many of these associations, especially those from the 3-year timepoint, remained significant after applying the Benjamini-Hochberg correction for multiple comparisons, which indicates robustness of these effects (see Table 1).

### 3.4 Path model analyses

Based on these correlations and on the fact that the CDS focuses mainly on pathogen-related disgust, we have further tested relationships between maternal disgust sensitivity (Total DS-R and Pathogen domain of TDDS), parity, and children's disgust sensitivity (Total CDS score) in path models. First, we used path models with the longitudinal pathway of maternal disgust sensitivity from 1T to 3Y, including all four measurement timepoints and parity, to assess its relation with children's disgust sensitivity.

**Figure 3:**
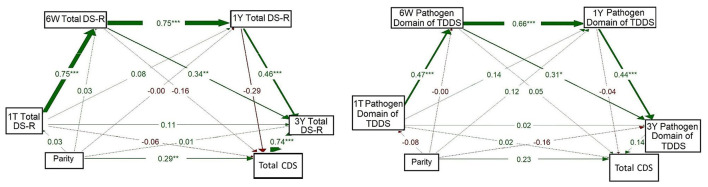
Full path models of concurrent and longitudinal associations between maternal pathogen disgust (Total DS-R on the left and Pathogen domain of TDDS on the right), parity, and children's disgust sensitivity (Total CDS). The abbreviations refer to various stages of pregnancy and postpartum: 1T, first trimester of pregnancy; 6W, 6 weeks postpartum; 1Y, 1 year postpartum; 3Y, 3 years postpartum; DS-R, Disgust Sensitivity-Revised; TDDS, Three Domains of Disgust Scale; CDS, Child Disgust Scale. The numbers (standardized path coefficients) and arrow widths indicate the strength of the associations. Asterisks indicate the level of statistical significance: *p* < 0.05 (*), *p* < 0.01 (**), and *p* < 0.001 (***).

The model on the left of Figure 3 shows the result for maternal disgust sensitivity measured using DS-R. In this model, Total DS-R had a strong positive association from 1T to 6W (β = 0.749, *p* < 0.001); this pattern continued from 6W to 1Y (β = 0.751, *p* < 0.001) and from 1Y to 3Y (β = 0.457, *p* < 0.001). Total DS-R at 6W was also positively associated with Total DS-R at 3Y (β = 0.339, *p* = 0.009). At 3Y, Total DS-R was strongly associated with CDS (β = 0.742, *p* < 0.001), but this was the only timepoint at which maternal disgust sensitivity had a significant effect on children's disgust sensitivity. Moreover, there emerged a direct path between parity and CDS (β = 0.287, *p* = 0.006); where multiparous women reported that their children had higher disgust sensitivity than the children of primiparous women. On the other hand, a comparison of the strength of these associations showed that the effect of maternal disgust sensitivity was more pronounced than the effect of parity. All paths from parity to Total DS-R at all timepoints were non-significant (βs = −0.001–0.03).

The model on the right of Figure 3 mirrored the model on the left but focused on disgust measured using the Pathogen domain of TDDS at the same timepoints. Again, a significant relationship was observed for the Pathogen domain from 1T to 6W (β = 0.474, *p* < 0.001), from 6W to 1Y (β = 0.660, *p* < 0.001), and from 1Y to 3Y (β = 0.437, *p* < 0.001). Moreover, the Pathogen domain at 6W was positively associated with the Pathogen domain at 3Y (β = 0.310, *p* = 0.015). No other pathways we examined, including direct paths from parity to disgust sensitivity of mothers or children, yielded significant results (βs = -0.16–0.23).

Given a large proportion of missing data at 6W and 1Y (see Table S3), we also constructed alternative models using only the 1T and 3Y timepoints, for which complete data were available from all participants. This allowed us to maintain the integrity of our analysis while providing a more comprehensive understanding of the results.

**Figure 4:**
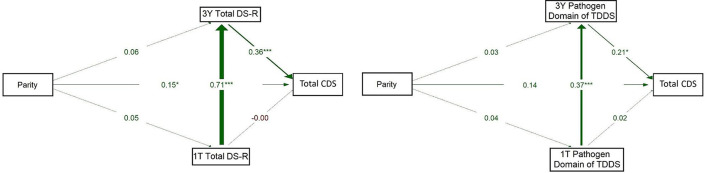
Path models of concurrent and longitudinal associations between maternal pathogen disgust sensitivity (Total DS-R on the left and Pathogen domain of TDDS on the right), parity, and children's disgust sensitivity (Total CDS). The abbreviations refer to various stages of pregnancy and postpartum: 1T, first trimester of pregnancy; 3Y, 3 years postpartum; DS-R, Disgust Sensitivity-Revised; TDDS, Three Domains of Disgust Scale; CDS, Child Disgust Scale. The numbers (standardized path coefficients) and arrow widths indicate the strength of the associations. Asterisks indicate the level of statistical significance: *p* < 0.05 (*) and *p* < 0.001 (***).

The model on the left of Figure 4 showed a strong positive association between 1T Total DS-R and 3Y Total DS-R (β = 0.710, *p* < 0.001) and from 3Y Total DS-R to CDS (β = 0.365, *p* < 0.001). The direct path from 1T Total DS-R to CDS was essentially null (β = −0.005, *p* = 0.963). A small but significant path emerged between parity and CDS (β = 0.149, *p* = 0.049), while other direct paths from parity were weak (βs = 0.05–0.06) and non-significant. This model indicated that parity had a direct positive association with children's disgust sensitivity, but no indirect effect through maternal disgust sensitivity. Additionally, 1T Total DS-R was not directly associated with children's disgust sensitivity; its influence emerged only indirectly, via its continuity into 3Y Total DS-R.

The model for the Pathogen domain of TDDS (on the right) showed a significant positive path from 1T Pathogen domain to 3Y Pathogen domain (β = 0.373, *p* < 0.001). Pathogen domain at 3Y significantly positively predicted CDS (β = 0.217, *p* = 0.015). The direct path from parity to CDS was non-significant (β = 0.143, *p* = 0.071) and paths from parity to the Pathogen domain at 1T and 3Y were also non-significant (β = 0.042, *p* = 0.609 and 0.031, *p* = 0.684, respectively). These results thus supported neither a direct nor indirect effect of parity on children's disgust sensitivity. Rather, they indicate that the 1T Pathogen domain was directly and positively associated with the 3Y Pathogen domain, which in turn showed a positive association with children's disgust sensitivity.

### 3.5 Correlations between maternal disgust sensitivity and maternal affective states

Kendall's correlation analyses were conducted to explore associations between maternal disgust sensitivity and various maternal affective states, including anxiety, perceived stress, and affectivity (positive and negative) at multiple time points (1T, 6W, 1Y, and 3Y).

#### 3.5.1 Anxiety and stress

Although STAI showed some significant correlation with maternal disgust sensitivity, this relationship did not remain significant after applying the Benjamini-Hochberg correction for multiple testing. In contrast, several significant positive correlations were found between maternal disgust sensitivity scores and maternal PSS. The most robust results after application of the Benjamini-Hochberg correction were observed in correlations between 1T PSS and both Total DS-R and the Pathogen domain of the TDDS. For more details, see Table 2 (for results regarding the DS-R subscales and the Sexual and Moral domains of TDDS, see Table S6).

**Table 2:** Correlation between maternal pathogen disgust sensitivity and maternal state anxiety (STAI) and perceived stress (PSS) across the measured periods.

**Variables**	**1T STAI**	**6W STAI**	**1Y STAI**	**3Y STAI**	**1T PSS**	**6W PSS**	**1Y PSS**	**3Y PSS**
	**Tau p**	**Tau p**	**Tau p**	**Tau p**	**Tau P**	**Tau p**	**Tau p**	**Tau p**
1T total DS-R	0.073 0.177	**0.170** **0.016**	0.000 0.997	0.054 0.313	0.178 0.004	0.237 0.001	0.070 0.352	0.061 0.264
1T pathogen TDDS	0.077 0.149	0.080 0.245	−0.118 0.112	0.031 0.561	0.133 0.031	0.125 0.074	0.017 0.822	0.030 0.582
6W total DS-R	0.039 0.568	0.016 0.819	−0.019 0.811	0.067 0.327	0.222 0.002	0.104 0.132	0.110 0.164	0.078 0.264
6W pathogen TDDS	**0.157** **0.024**	0.119 0.085	−0.012 0.880	0.075 0.276	0.272 0.000	**0.168** **0.016**	0.105 0.186	0.087 0.212
1Y total DS-R	0.011 0.879	−0.086 0.277	−0.032 0.660	0.026 0.721	0.200 0.007	0.055 0.491	0.069 0.346	0.063 0.401
1Y pathogen TDDS	−0.01 0.888	−0.028 0.721	−0.056 0.449	−0.048 0.515	0.157 0.035	0.034 0.667	0.053 0.472	−0.009 0.900
3Y total DS-R	0.078 0.143	0.049 0.470	−0.089 0.229	0.088 0.094	0.192 0.002	0.100 0.147	−0.006 0.938	0.072 0.178
3Y pathogen TDDS	0.019 0.726	0.050 0.474	−0.124 0.098	0.054 0.308	0.154 0.013	0.118 0.093	−0.032 0.668	0.040 0.459

The abbreviations refer to stages of pregnancy and postpartum: 1T, first trimester of pregnancy; 6W, 6 weeks postpartum; 1Y, 1 year postpartum; 3Y, 3 years postpartum. Significant correlations are bolded. Those correlations which remained significant after applying Benjamini-Hochberg correction for multiple testing are underlined, FDR was set at 0.1.

#### 3.5.2 Positive and negative affectivity

Maternal disgust sensitivity showed generally negative correlations with PA; in particular, higher Total DS-R scores at 1T were significantly associated with lower PA at 6W (τ = −0.163, *p* = 0.021) and higher Total DS-R scores at 1Y were significantly associated with lower PA at 3Y (τ = −0.149, *p* = 0.048). Still, none of these correlations remained significant after correction for multiple testing. Several positive correlations were also observed between maternal disgust sensitivity and NA at 1T but only the association between the Pathogen domain at 6W and NA at 1T remained significant after correction for multiple testing (τ = 0.212, *p* = 0.003). For further details, see Table 3 (for results regarding the DS-R subscales and Sexual and Moral domains of TDDS, see Table S7).

**Table 3:** Correlation analysis of maternal disgust sensitivity and maternal positive and negative affectivity.

**Variables**	**1T PA**	**6W PA**	**1Y PA**	**3Y PA**	**1T NA**	**6W NA**	**1Y NA**	**3Y NA**
	**Tau p**	**Tau p**	**Tau p**	**Tau p**	**Tau P**	**Tau p**	**Tau p**	**Tau p**
1T total DS-R	−0.021 0.734	**−0.163** **0.021**	−0.106 0.160	−0.030 0.584	0.103 0.102	0.130 0.067	−0.023 0.760	0.065 0.230
1T pathogen TDDS	0.045 0.470	−0.013 0.849	0.020 0.784	0.013 0.816	0.077 0.216	0.111 0.111	−0.072 0.335	0.058 0.285
6W total DS-R	−0.079 0.260	−0.076 0.271	−0.125 0.113	−0.066 0.349	**0.145** **0.038**	0.032 0.645	0.034 0.663	0.045 0.523
6W pathogen TDDS	−0.053 0.451	−0.086 0.215	−0.025 0.755	−0.043 0.543	0.212 0.003	0.089 0.196	0.017 0.833	0.058 0.410
1Y total DS-R	−0.066 0.374	−0.062 0.434	−0.131 0.075	**−0.149** **0.048**	**0.158** **0.034**	−0.046 0.565	−0.003 0.969	0.008 0.911
1Y pathogen TDDS	−0.067 0.370	0.055 0.490	0.005 0.946	−0.037 0.623	0.101 0.175	−0.087 0.273	−0.098 0.185	−0.092 0.220
3Y total DS-R	−0.025 0.684	−0.103 0.138	−0.092 0.211	−0.035 0.521	**0.145** **0.019**	0.042 0.545	−0.077 0.299	0.094 0.081
3Y pathogen TDDS	0.017 0.782	−0.043 0.542	0.063 0.401	−0.018 0.743	0.122 0.050	0.048 0.496	−0.134 0.072	0.079 0.144

The abbreviations refer to various stages of pregnancy and postpartum: 1T, first trimester of pregnancy; 6W, 6 weeks postpartum; 1Y, 1 year postpartum; 3Y, 3 years postpartum. Significant correlations are bolded. Those correlations which remained significant after applying Benjamini-Hochberg correction for multiple testing are underlined, FDR was set at 0.1.

### 3.6 Correlations between maternal affective states and disgust sensitivity in children

Kendall's correlation analyses were also carried out to examine associations between maternal affective states (including anxiety, perceived stress, and affectivity) at all timepoints and children's disgust sensitivity at 3 years postpartum.

Overall, few significant associations were observed. PSS at 6W showed a week but significant positive correlation with CDS Affect subscale (τ = 0.147, *p* = 0.040), suggesting that higher early maternal stress may be related to increased disgust affect in children. Regarding maternal PA, significant negative correlations were found with CDS scores. Specifically, PA at 1Y was negatively associated with both the CDS Affect subscale (τ = −0.173, *p* = 0.022) and Total CDS scores (τ = −0.150, *p* = 0.048), while PA at 6W postpartum was related only to CDS Avoidance subscale (τ = −0.143, *p* = 0.046). These findings indicate that lower maternal PA in the postpartum period may be associated with higher levels of children's disgust sensitivity. No significant correlations were found between the STAI scores at any timepoint and CDS scores (*p* > 0.05) and similarly, maternal NA showed no significant associations with CDS scores at any of the timepoints.

Although a few associations did reach the level of statistical significance (*p* < 0.05), none remained significant after applying the Benjamini-Hochberg correction for multiple testing, suggesting that these findings should be interpreted with caution (see Table S8).

### 3.7 Path model analyses: the role of maternal affective states

Based on the results of these correlation analyses, we used path model analysis to investigate the role of maternal affective states (especially NA at 1T and PSS at 1T) in associations between parity, maternal disgust (Total DS-R and Pathogen domain of TDDS), and children's disgust sensitivity (Total CDS). For this purpose, we used models with 1T and 3Y measurements, for which complete data were available from all participants.

**Figure 5:**
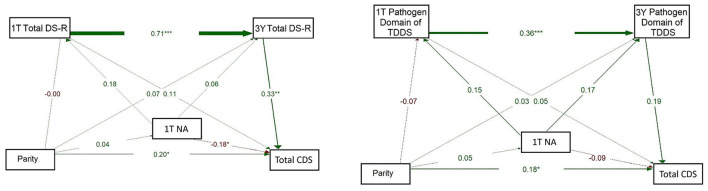
Path models of concurrent and longitudinal associations between maternal pathogen disgust sensitivity (Total DS-R on the left and Pathogen of TDDS on the right), maternal negative affectivity (1T NA), parity, and children's disgust sensitivity (Total CDS). The abbreviations refer to various stages of pregnancy and postpartum: 1T, first trimester of pregnancy; 3Y, 3 years postpartum; DS-R, Disgust Sensitivity-Revised; TDDS, Three Domains of Disgust Scale; CDS, Child Disgust Scale; NA, Negative Affectivity. The numbers (standardized path coefficients) and arrow widths indicate the strength of the associations. Asterisks indicate the level of statistical significance: *p* < 0.05 (*), *p* < 0.01 (**), and *p* < 0.001 (***).

The model on the left of Figure 5 explored whether Total DS-R score and NA at 1T, and Total DS-R at 3 years, were associated with CDS, whereby parity was treated as the independent variable. As in previous models, Total DS-R scores at 1T significantly predicted Total DS-R at 3Y (β = 0.705, *p* = 0.001). Total DS-R score at 3Y was significantly associated with CDS (β = 0.327, *p* = 0.007), suggesting that higher maternal disgust sensitivity at this timepoint predicts higher disgust sensitivity in children. Maternal NA at 1T was positively associated with Total DS-R score at 1T (β = 0.182, *p* = 0.052). Maternal NA at 1T was also negatively but significantly associated with CDS (β = −0.180, *p* = 0.037), suggesting a potentially suppressive effect. The path from parity to NA at 1T was weak and non-significant (β = 0.038, *p* = 0.693) but the direct effect of parity on CDS proved significant (β = 0.202, *p* = 0.017). Altogether, this model indicates that maternal NA and Total DS-R are related early in pregnancy but that children's disgust sensitivity is better predicted by maternal disgust sensitivity at 3Y than by parity or NA directly. These findings also suggest that parity is associated with children's disgust sensitivity independently of the effects of NA or maternal disgust sensitivity measured using Total DS-R.

The model on the right of Figure 5 evaluated the influence of maternal NA at 1T and the Pathogen domain of TDDS at 1T and 3Y, on children's disgust sensitivity, with parity as an independent variable. Pathogen domain scores showed continuity over time, with a significant positive association from 1T to 3Y (β = 0.357, *p* = 0.001). However, the Pathogen domain was not significantly associated with CDS at either 1T (β = 0.054, *p* = 0.586) or 3Y (β = 0.189, *p* = 0.059). Similarly, the path from 1T NA to 3Y Pathogen domain was not significant (β = 0.169, *p* = 0.053) and neither was the path to CDS, which was negative and non-significant (β = −0.086, *p* = 0.362). Nevertheless, a small but significant positive association was observed between parity and CDS (β = 0.185, *p* = 0.044), where multiparous mothers reported higher levels of disgust sensitivity in their children. Still, parity was not significantly associated with the Pathogen domain at 1T (β = −0.065, *p* = 0.487) or with NA at 1T directly (β = 0.048, *p* = 0.612). Overall, this model indicates that, independently of the Pathogen domain of TDDS or NA, parity may influence child disgust sensitivity.

**Figure 6:**
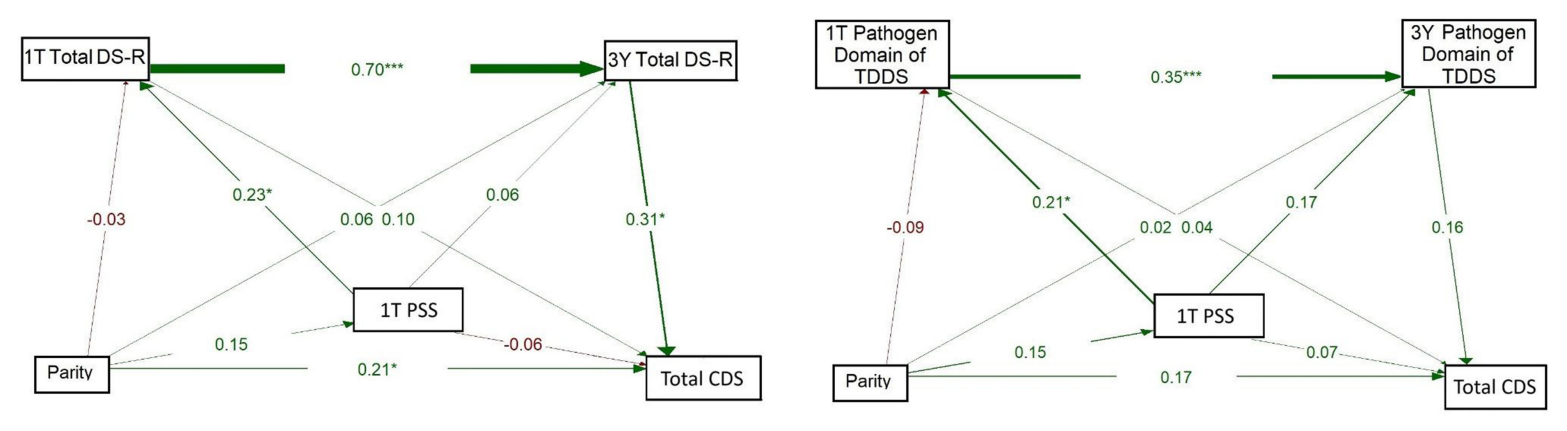
Path models of concurrent and longitudinal associations between maternal pathogen disgust sensitivity (Total DS-R on the left and Pathogen domain of TDDS on the right), maternal perceived stress (1T PSS), parity, and children's disgust sensitivity (Total CDS). The abbreviations refer to various stages of pregnancy and postpartum: 1T, first trimester of pregnancy; 3Y, 3 years postpartum; DS-R, Disgust Sensitivity-Revised; TDDS, Three Domains of Disgust Scale; CDS, Child Disgust Scale; PSS, Perceived Stress Scale. The numbers (standardized path coefficients) and arrow widths indicate the strength of the associations. Asterisks indicate the level of statistical significance: *p* < 0.05 (*) and *p* < 0.001 (***).

The model on the left of Figure 6 explored whether Total DS-R and PSS at 1T and Total DS-R at 3Y were associated with child disgust, with parity as the independent variable. The analysis confirmed a strong association between Total DS-R scores at both 1T and 3Y (β = 0.703, *p* = 0.001). Maternal PSS at 1T was significantly associated with Total DS-R at 1T (β = 0.227, *p* = 0.015) and Total DS-R at 3Y was positively and significantly associated with CDS (β = 0.311, *p* = 0.012). Nevertheless, the direct path from 1T PSS to CDS was negative and non-significant (β = −0.064, *p* = 0.473). Parity showed no significant associations with 1T Total DS-R (β = −0.032, *p* = 0.729), 1T PSS (β = 0.152, *p* = 0.105), or 3Y Total DS-R (β = 0.058, *p* = 0.384), but it was significantly and positively associated with CDS (β = 0.212, *p* = 0.015). These findings indicate that maternal disgust sensitivity may be shaped by early stress and that it is stable over time, which in turn may influence children's disgust sensitivity. And while parity did not influence children's disgust sensitivity indirectly via maternal disgust sensitivity or stress, it had a direct effect: being multiparous was associated with reporting higher scores of CDS. Additionally, the findings suggest that while early maternal stress may contribute to maternal disgust sensitivity in early pregnancy, and this sensitivity seems relatively stable over time, the overall indirect pathway from parity to children's disgust sensitivity through this circuit is weak.

The model on the right of Figure 6 examined whether PSS and Pathogen domain of TDDS at 1T and at 3Y mediated the relationship between parity and children's disgust sensitivity. The results indicated a significant positive association between the Pathogen domain at 1T and at 3Y (β = 0.347, *p* = 0.001). Maternal PSS at 1T was positively associated with the Pathogen domain at 1T (β = 0.213, *p* = 0.023) but the direct path from 1T PSS to CDS was not significant (β = 0.068, *p* = 0.474). Additionally, Pathogen domain at 3Y was positively but non-significantly associated with CDS (β = 0.160, *p* = 0.108). No significant direct effects were found between parity and either Pathogen domain at T1 (β = −0.090, *p* = 0.333), 1T PSS (β = 0.153, *p* = 0.101) or CDS (β = 0.173, *p* = 0.061). These findings suggest that while early maternal stress may influence the Pathogen domain, and the Pathogen domain shows developmental continuity, the strength of the overall indirect pathway from parity to children's disgust sensitivity via these maternal factors is limited. Additionally, neither stress nor pathogen disgust measured using the Pathogen domain of TDDS seem to affect children's disgust sensitivity.

## 4 Discussion

The main aim of this study was to assess whether maternal disgust sensitivity measured from the first trimester of pregnancy to 3 years postpartum predicts children's disgust sensitivity and whether the relationship is affected by parity and maternal psychological factors. Across multiple measures and at different timepoints, our results showed significant positive correlations between maternal disgust sensitivity and children's disgust sensitivity. Path analyses further highlighted this relationship: they showed that maternal disgust sensitivity at each timepoint significantly predicted maternal disgust at the next point, and that higher maternal disgust at 3 years postpartum significantly predicted higher levels of children's disgust sensitivity. Moreover, perceived stress (significantly) and negative affectivity (close to significance level) in the first trimester positively predicted maternal disgust sensitivity in the first trimester. Importantly, while multiparity was also associated with increased children's disgust sensitivity, its effect seems to be direct and not mediated by maternal disgust sensitivity or mother's psychological factors.

### 4.1 Maternal disgust sensitivity predicts disgust sensitivity in the child

Our findings show that maternal disgust sensitivity significantly positively predicted children's disgust sensitivity in our sample. Although we have observed significant correlations between children's disgust sensitivity (both Avoidance and Affect subscales of the CDS as well as the total score) and maternal disgust sensitivity at multiple time points, path analyses indicated that these associations were driven mainly by the continuity of maternal disgust sensitivity over time, with the strongest effect emerging between disgust sensitivity in the mother and child measured at 3 years postpartum. This may be partly related to the developmental milestones which children typically reach around the age of three, when they begin to engage in cooperative activities with peers and participate in pretend play (Lillard et al., 2011). These activities often involve increased physical contact with other children and shared objects, which can raise the likelihood of exposure to potential pathogens. As a result, mothers may start to experience more intense feelings of disgust. This heightened sensitivity can then be reflected in their behaviour, which children observe and internalise, potentially lead to increased disgust sensitivity observed in the children themselves.

Previous studies also show that parents, and especially those of young children, express disgust more intensively and through multiple models of expression, including face, voice, and gestures (Stevenson et al., 2010; Oaten et al., 2014). This could be explained by the need to teach young children to recognise and react to disgusting stimuli. Stevenson et al. (2014) have shown that higher parental disgust predicted that children would look longer at disgusting images when a disgust vocalisation was played to them. These findings indirectly support our results, which showed a significant positive association between disgust sensitivity in mothers and children. This suggests that specific behavioural patterns in parent–child interactions may serve as underlying mechanisms for the transmission of disgust sensitivity. Moreover, findings show that higher disgust sensitivity in mothers, as measured by the DS-R, in combination with their children's disgust sensitivity, as measured by the CDS, predicted disgust-related learning in children (Olatunji and Viar-Paxton, 2020). This learning was demonstrated in a task where children rated their level of disgust toward an animal after being given dirt- and disease-related information about it by the experimenter.

Given that certain differences in disgust sensitivity results measured with the DS-R and TDDS have been previously observed in a number of studies, we used both questionnaires and compared their results. Like in previous studies (Dlouhá and Kanková, 2024; Dlouhá et al., 2024; Kanková et al., 2024; Takács et al., 2025a), our results showed stronger and more statistically significant effects in disgust sensitivity measured with the DS-R. Our findings add to the body of evidence which suggests that the TDDS questionnaire, particularly its Pathogen domain, may not be sufficiently sensitive to capture maternal disgust sensitivity associated with the adaptive changes related to motherhood. Another possible explanation for the differences observed between the two questionnaires is that the CDS was specifically designed to assess disgust sensitivity in children, using age-appropriate items adapted from the DS-R (Viar-Paxton et al., 2015). Since the CDS may better capture how children experience and express disgust, it could more accurately reflect the dynamics between maternal disgust sensitivity (measured by the DS-R) and children's responses, which may explain the stronger effects observed in our study.

Regarding the other domains of TDDS, the Moral domain showed no statistically significant relationship with children's disgust sensitivity, except for a correlation between the Moral domain at 1T and the CDS Affect subscale. In contrast, correlation analyses revealed multiple positive and statistically significant associations between the Sexual domain and children's disgust sensitivity at all measured timepoints. In fact, higher levels of sexual disgust have been linked to political conservatism (Billingsley et al., 2018; van Leeuwen et al., 2017), and it has also been observed in individuals with higher levels of neuroticism and conscientiousness (Tybur and de Vries, 2013). These characteristics may lead to parenting styles that contribute to increased disgust sensitivity in children. Moreover, mothers with a high sexual disgust sensitivity may create an environment where physical boundaries are emphasised and topics related to the body are treated as uncomfortable. These cues, be they verbal or non-verbal, conscious or subconscious, could be internalised by the child and shape broader disgust sensitivity through social learning and behavioural modelling.

Finally, it is important to note that children's disgust sensitivity was assessed in our study through the mothers' reports and although the participating mothers were explicitly instructed to answer how their child would really behave and not how they would want them to behave, the reported children's disgust sensitivity scores may have been nevertheless influenced by the mothers' own disgust sensitivity. Moreover, while mothers can easily report on their child's behaviour (the CDS Avoidance subscale), they may be less accurate when it comes to identifying their children's emotional experience of disgust (CDS Affect subscale). Interestingly, while parity had a significant effect on children's disgust sensitivity, it showed no association with maternal disgust, which suggests that at least part of the observed children's disgust sensitivity reflects variance independently of self-reported maternal disgust sensitivity.

### 4.2 The effect of maternal parity on disgust sensitivity in the child

Compared to primiparous women, multiparous women consistently reported higher levels of children's disgust sensitivity across both the total score and the Affect subscale. Moreover, contrary to previous studies which hypothesised an indirect effect mediated by maternal disgust sensitivity (Dlouhá et al., 2023; Kanková et al., 2023b), our findings showed that the effect of parity was direct, as we found no statistically significant associations between parity and maternal disgust. One possible explanation for this direct effect may lie in the influence of older siblings in multiparous households. Older siblings may serve as secondary models of disgust-related behaviours, as actors who reinforce the transmission of disgust sensitivity. It has been observed that siblings play a key role in the development of their younger siblings' emotional understanding (Kramer, 2014)—and this could play a role in the effectivity of disgust learning as well. A second explanation is that larger households, which are more common among multiparous families, may involve greater exposure to environmental pathogens: when more individuals share the living space, and especially when this includes young children who are still acquiring hygiene habits, the overall microbial load in the household environment tends to be higher. Moreover, the presence of older siblings, who may bring pathogens from school or other social settings, further increases the likelihood of pathogen exposure. As a result, heightened disgust sensitivity may develop in younger siblings as a way to mitigate these increased health risks through greater aversion to unclean or potentially infectious stimuli.

### 4.3 The effect of maternal affective states on disgust sensitivity in the child

It has repeatedly been reported that higher disgust sensitivity is associated with anxiety (Olatunji et al., 2010, 2017; Takács et al., 2025a) and anxiety-related disorders (Davey et al., 2008; Olatunji et al., 2007a, 2010). In our study, maternal anxiety showed modest positive associations with maternal disgust sensitivity at the early timepoints, particularly in the first trimester of pregnancy and shortly after birth, with stronger links to contamination-related disgust. However, after correction for multiple testing, these associations were relatively weak and non-significant. Importantly, maternal anxiety was not significantly associated with children's disgust sensitivity at any point, which suggests that while anxiety may modestly influence maternal emotional responses to disgust-eliciting stimuli, it does not play a meaningful role in the development of disgust sensitivity in children.

On the other hand, maternal perceived stress particularly in early pregnancy, played an indirect role in shaping children's disgust sensitivity by influencing maternal disgust sensitivity. Higher scores of PSS in the first trimester were significantly associated with greater maternal pathogen-related disgust sensitivity, as path analyses revealed. A similar trend was observed for NA in the first trimester; however, this effect did not reach statistical significance, although it approached the conventional threshold (*p* = 0.052). Although early maternal stress did not directly relate to children's disgust sensitivity, it apparently contributed to elevated maternal disgust sensitivity that persisted and ultimately influenced the child. It is possible that maternal stress triggers an increase in disgust sensitivity in the mother during the early stages of pregnancy (which probably serves adaptive purposes early on). However, its effects may be later mitigated by compensatory biological mechanisms (such as hormonal regulation) or psychological strategies (such as adaptive coping), which could explain why we did not observe these associations after the first trimester. These mechanisms may help prevent the mother's disgust sensitivity from escalating, which could possibly lead to adverse outcomes, such as mental health issues. In the context of these results relating to maternal stress, we could extend the interpretation to include prenatal programming; the idea that mother's psychological experiences during pregnancy affect the child's developmental outcomes (Barker, 1990; Bush, 2024). For that, however, we would expect to observe a direct effect of maternal psychological factors in early pregnancy on children's disgust sensitivity, which was, as mentioned above, not present in our analyses.

As for 1T NA, although it does not significantly influence the child's disgust sensitivity indirectly through maternal disgust sensitivity, it does have a direct, statistically significant negative effect. This may suggest that higher maternal NA early in pregnancy leads to heightened emotional reactivity and, potentially, greater reliance on avoidant or protective behaviors as compensatory strategies to regulate these negative emotional states. These behaviors may, in turn, reduce the child's exposure to disgust-related stimuli, thereby dampening the development of child disgust sensitivity. Nevertheless, this interpretation should be treated with caution, as the observed effect may instead reflect maternal reporting bias among mothers with higher NA in our study, rather than the child's emotional development.

Regarding these results, however, it should be noted that our participants were recruited mostly during the COVID-19 pandemic, a period associated with elevated anxiety, stress, and negative affectivity (Turna et al., 2021; Santos et al., 2022). A recent study has indicated that pregnant women experienced higher levels of disgust during the pandemic than those pregnant prior to it (Kanková et al., 2023b). It is thus possible that the heightened levels of maternal stress, negative affectivity, and disgust observed in the first trimester were influenced by the pandemic and affected the patterns found in our study. Given the wide time span of data collection at 1T (June 2019–June 2021) and the subsequent longitudinal follow-up, it would be difficult to statistically isolate the direct impact of the pandemic, as it influenced individual participants at different stages of testing and to varying degrees, from strict lockdowns to periods of relative relaxation. Therefore, we interpret this factor mainly theoretically, suggesting that pandemic-related stress may have amplified activation of the behavioural immune system. These elevated maternal factors may have then in turn influenced the development of children's disgust sensitivity as part of a broader adaptive response to a perceived increased pathogen threat. Still, from the evolutionary perspective, high levels of stress were probably a constant feature of the ancestral environment for pregnant women or caregiving mothers. In this sense, it is also possible that the COVID-19 pandemic may be a particularly relevant modern parallel that offered insights into how such conditions may have affected emotional interactions between mothers and children and developmental outcomes in the past.

### 4.4 Strengths and limitations

This study offers several contributions to the literature. To the best of our knowledge, this is the first study to investigate maternal disgust sensitivity, affective states, and parity as predictors of children's disgust sensitivity in a longitudinal, multivariate model.

Our study also has several limitations. Aside from the abovementioned issue of assessment of children's disgust sensitivity via maternal report, the use of self-report measures to assess maternal disgust sensitivity is also a methodological limitation. Our findings should therefore be interpreted with caution. Future research would benefit from incorporating experimental approaches that extend beyond self-report, including physiological measures of responses related to disgust sensitivity. Moreover, research should consider assessments across multiple sensory modalities, such as vision, olfaction, and touch.

The second limitation of this study is the missing data at 6W and 1Y timepoints. Women were recruited during the first trimester of pregnancy and invited to participate in a follow-up study that would include assessments at multiple stages after birth. Due to insufficient response rates at 6W and 1Y, we introduced a financial incentive to encourage participation in the 3-year postpartum follow-up, at which point children's disgust sensitivity was also assessed for the first time. Nevertheless, path models based on the smaller subset of women with complete data and those based on the larger group of participants with data at 1T and 3Y yielded similar conclusions and comparable effect sizes.

## 5 Conclusion

Our findings suggest that disgust sensitivity in 3-year-old children may be in part shaped by maternal disgust sensitivity, which has continuously evolved during pregnancy and after birth. Maternal emotional states such as perceived stress and, to a lesser degree, negative affectivity during pregnancy may also play an important role in this process. Aside from this, higher levels of children's disgust sensitivity were reported by multiparous mothers, which indicates that the presence of older siblings might contribute to individual differences in early affective development. Moreover, older siblings may serve as behavioural models or contribute to increasing pathogen load in the home environment. Our results highlight the developmental continuity of maternal disgust sensitivity from pregnancy until 3 years postpartum and its association with the early development of the child's own disgust sensitivity.

## Data Availability

The datasets presented in this study can be found in online repositories. The names of the repository/repositories and accession number(s) can be found at: 10.6084/m9.figshare.29590139.
