# Supplementary material for: The impact of maternal disgust sensitivity from pregnancy until 3 years postpartum on the early development of disgust sensitivity in the child
Source: Front Psychol. 2025 Nov 27;16:1680260. doi: 10.3389/fpsyg.2025.1680260 (PMC12695615; doi:10.3389/fpsyg.2025.1680260)
Supplement: Supplementary file 1 [file Supplementary_file_1.docx]

**The impact of maternal disgust sensitivity from pregnancy until three years** **postpartum on the early development of disgust sensitivity in the child**

**Supplementary Materials**

Table S1. Characteristics of the samples S1+S2, S1, and S2.

|  | | **S1+S2** | **S1** | **S2** |
| --- | --- | --- | --- | --- |
| **n** | | 163 | 42 | 121 |
| **Age** | Mean (SD)  Range | 32.2 (4.1)  21–42 | 31.8 (3.8)  23–40 | 32.3 (4.3)  21–42 |
| **Gestational age (in days)** | Mean (SD)  Range  Missing data, n | 278 (9.6)  235–304  4 | 278 (7.6)  264–295  0 | 277 (10.3)  235–304  4 |
| **Pre-pregnancy weight** | Mean (SD)  Range  Missing data, n | 65.7 (12.8)  45.5–106  1 | 64.3 (13.2)  45.5–106  0 | 66.2 (12.7)  47.0–105  1 |
| **Parity** | Primipara, n (%)  Multipara, n (%) | 97 (59.5%)  66 (40.5%) | 29 (69.0%)  13 (31.0%) | 68 (56.2%)  53 (43.8%) |
| **Child sex** | Girl, n (%)  Boy, n (%)  Missing data, n | 87 (53.7%)  75 (46.3%)  1 | 27 (64.3%)  15 (35.7%)  0 | 60 (50.0%)  60 (50.0%)  1 |
| **Birth weight (in grams)** | Mean (SD)  Range  Missing data, n | 3398 (463)  1800–4700  1 | 3390 (464)  2360–4620  0 | 3400 (464)  1800–4700  1 |
| **Preterm birth (<259 days) or low birth weight (<2500 g)** | Yes, n (%)  No, n (%)  Missing data, n | 8 (4.9%)  154 (95.1%)  1 | 1 (2.4%)  41 (97.6%)  0 | 7 (5.8%)  113 (94.2%)  1 |
| **Delivery mode** | Spontaneous vaginal, n (%)  Operative vaginal, n (%)  Acute caesarean section, n (%)  Planned caesarean section, n (%) | 107 (65.6%)  3 (1.8%)  23 (14.1%)  30 (18.4%) | 26 (61.9%)  1 (2.4%)  6 (14.3%)  9 (21.4%) | 81 (66.9%)  2 (1.7%)  17 (14.0%)  21 (17.4%) |
| **Education level** | Elementary school or apprenticeship, n (%)  Secondary school, n (%)  University, n (%)  Missing data, n | 3 (1.8%)  33 (20.4%)  126 (77.8%)  1 | 0 (0.0%)  4 (9.5%)  38 (90.5%)  0 | 3 (2.5%)  29 (24.2%)  88 (73.3%)  1 |
| **Monthly household**  **income (in thousands**  **CZK)** | <30, n (%)  30–45, n (%)  46–60, n (%)  61–75, n (%)  76–90, n (%)  >90, n (%)  Missing data, n | 16 (9.9%)  30 (18.5%)  45 (27.8%)  29 (17.9%)  24 (14.8%)  18 (11.1%)  1 | 0 (0.0%)  4 (9.8%)  9 (22.0%)  11 (26.8%)  10 (24.4%)  7 (17.1%)  1 | 16 (13.2%)  26 (21.5%)  36 (29.8%)  18 (14.9%)  14 (11.6%)  11 (9.1%)  0 |

Table S2: Child Disgust Scale (CDS) - descriptive statistics

| Variables | N | Mean | SD | Median | Min | Max | Cronbach’s α |
| --- | --- | --- | --- | --- | --- | --- | --- |
| Total CDS | 152 | 9.01 | 4.66 | 9 | 0 | 21 | 0.80 |
| Affect CDS | 153 | 1.62 | 1.72 | 1 | 0 | 7 | 0.68 |
| Avoidance CDS | 157 | 7.31 | 3.53 | 7 | 0 | 16 | 0.73 |

Table S3: Maternal disgust (DS-R and TDDS) across the measured periods - descriptive statistics

| Variables | N | Mean | SD | Median | Min | Max | Cronbach’s α |
| --- | --- | --- | --- | --- | --- | --- | --- |
| 1T Total DS-R | 159 | 51.81 | 13.86 | 51 | 21 | 83 | 0.83 |
| 1T Core DS-R | 159 | 27.16 | 7.07 | 26 | 10 | 44 | 0.69 |
| 1T Contamination DS-R | 159 | 7.86 | 3.68 | 8 | 0 | 17 | 0.60 |
| 1T Animal-Reminder DS-R | 159 | 16.79 | 6.18 | 17 | 2 | 32 | 0.74 |
| 1T Pathogen TDDS | 162 | 19.23 | 7.59 | 19 | 2 | 40 | 0.75 |
| 1T Sexual TDDS | 162 | 24.67 | 10.24 | 27 | 0 | 42 | 0.75 |
| 1T Moral TDDS | 162 | 22.56 | 8.09 | 23 | 2 | 42 | 0.89 |
| 6W Total DS-R | 98 | 50.10 | 13.94 | 50.5 | 21 | 87 | 0.85 |
| 6W Core DS-R | 98 | 26.02 | 7.21 | 26 | 8 | 41 | 0.75 |
| 6W Contamination DS-R | 98 | 7.64 | 3.74 | 7.5 | 0 | 17 | 0.63 |
| 6W Animal-Reminder DS-R | 98 | 16.44 | 6.03 | 17 | 2 | 30 | 0.75 |
| 6W Pathogen TDDS | 97 | 21.76 | 7.40 | 22 | 6 | 39 | 0.80 |
| 6W Sexual TDDS | 97 | 16.40 | 7.51 | 15 | 2 | 34 | 0.73 |
| 6W Moral TDDS | 97 | 24.21 | 10.19 | 25 | 0 | 42 | 0.90 |
| 1Y Total DS-R | 85 | 49.05 | 13.25 | 47 | 17 | 92 | 0.84 |
| 1Y Core DS-R | 85 | 25.62 | 6.85 | 25 | 8 | 46 | 0.73 |
| 1Y Contamination DS-R | 85 | 7.13 | 3.28 | 7 | 0 | 16 | 0.54 |
| 1Y Animal-Reminder DS-R | 85 | 16.29 | 5.99 | 17 | 0 | 30 | 0.74 |
| 1Y Pathogen TDDS | 85 | 21.12 | 7.60 | 20 | 2 | 41 | 0.81 |
| 1Y Sexual TDDS | 85 | 14.71 | 8.14 | 12 | 1 | 37 | 0.79 |
| 1Y Moral TDDS | 85 | 23.31 | 11.37 | 26 | 0 | 42 | 0.93 |
| 3Y Total DS-R | 163 | 50.99 | 14.06 | 51 | 16 | 93 | 0.85 |
| 3Y Core DS-R | 163 | 26.55 | 7.32 | 27 | 11 | 47 | 0.75 |
| 3Y Contamination DS-R | 163 | 7.32 | 3.30 | 7 | 0 | 16 | 0.49 |
| 3Y Animal-Reminder DS-R | 163 | 17.12 | 6.24 | 17 | 2 | 32 | 0.76 |
| 3Y Pathogen TDDS | 161 | 23.43 | 7.81 | 23 | 6 | 42 | 0.78 |
| 3Y Sexual TDDS | 161 | 16 | 7.82 | 15 | 1 | 37 | 0.74 |
| 3Y Moral TDDS | 160 | 25.84 | 10.74 | 28 | 0 | 42 | 0.90 |

The abbreviations refer to various stages of pregnancy and postpartum recovery: 1T – first trimester of pregnancy, 6W – six weeks postpartum, 1Y – one year postpartum, and 3Y – three years postpartum.

Table S4: Maternal affective states across the measured periods - descriptive statistics

| Variables | N | Mean | SD | Median | Min | Max | Cronbach’s α |
| --- | --- | --- | --- | --- | --- | --- | --- |
| 1T STAI | 161 | 10.99 | 3.39 | 10 | 6 | 21 | 0.86 |
| 6W STAI | 98 | 9.87 | 3.05 | 9 | 6 | 18 | 0.84 |
| 1Y STAI | 85 | 10.04 | 3.26 | 10 | 6 | 20 | 0.86 |
| 3Y STAI | 163 | 11.47 | 3.34 | 11 | 6 | 22 | 0.86 |
| 1T PSS | 121 | 12.89 | 6.09 | 14 | 0 | 33 | 0.87 |
| 6W PSS | 96 | 13.46 | 6.07 | 13 | 0 | 30 | 0.89 |
| 1Y PSS | 85 | 15.32 | 6.45 | 15 | 0 | 31 | 0.90 |
| 3Y PSS | 158 | 17.30 | 6.08 | 17.5 | 2 | 30 | 0.87 |
| 1T PA | 120 | 30.36 | 7.30 | 31 | 14 | 46 | 0.89 |
| 6W PA | 96 | 32.11 | 6.94 | 33 | 13 | 45 | 0.87 |
| 1Y PA | 85 | 32.46 | 7.38 | 34 | 13 | 46 | 0.89 |
| 3Y PA | 157 | 31.57 | 6.80 | 32 | 13 | 47 | 0.86 |
| 1T NA | 120 | 18.20 | 6.58 | 17 | 10 | 46 | 0.89 |
| 6W NA | 96 | 16.93 | 5.74 | 16 | 10 | 36 | 0.88 |
| 1Y NA | 85 | 18.11 | 6.55 | 17 | 10 | 39 | 0.90 |
| 3Y NA | 157 | 19.80 | 7.21 | 18 | 10 | 45 | 0.90 |

The abbreviations refer to various stages of pregnancy and postpartum: 1T – first trimester of pregnancy, 6W – six weeks postpartum, 1Y – one year postpartum, and 3Y – three years postpartum.

Table S5: Associations between demographic variables and child disgust

| Variables | Total CDS | | Affect CDS | | Avoidance CDS | |
| --- | --- | --- | --- | --- | --- | --- |
|  | Tauª/Mann-  Whitney U | p | Tauª/Mann-  Whitney U | p | Tauª/Mann-  Whitney U | p |
| Maternal age 3^rd^ year postpartum | -0.045ª | 0.408 | -0.079ª | 0.149 | -0.002ª | 0.971 |
| Education | 1868 | 0.589 | 1808 | 0.280 | 2070 | 0.841 |
| Parity* | **2208** | **0.043** | **2233** | **0.032** | 2484 | 0.097 |
| Child sex | 2562 | 0.292 | 2838 | 0.873 | 2814 | 0.434 |
| Delivery mode | 2403 | 0.394 | 2581 | 0.787 | 2562 | 0.328 |
| Recruitment place | 1942 | 0.270 | 2170 | 0.819 | 1980 | 0.145 |

Significant correlations are bolded. The correlations did not remain significant after applying the Benjamini-Hochberg correction for multiple testing, FDR was set at 0.1. The variables were coded as follows: Education (0 = high school with graduation or lower, 1 = university), Parity (0 = primiparae, 1 = multiparae), Child sex (0 = female, 1 = male), Delivery Mode (0 = other than spontaneous vaginal, 1 = spontaneous vaginal), and Recruitment Place (0= private gynecological clinics, 1 = general university hospital). *Children of multiparae women had higher total CDS score and Affect CDS score.

Table S6: Correlation between maternal disgust and maternal state anxiety (STAI) and Perceived Stress (PSS) across the measured periods

| Variables | Maternal Affective States | | | | | | | |
| --- | --- | --- | --- | --- | --- | --- | --- | --- |
|  | 1T STAI | 6W STAI | 1Y STAI | 3Y STAI | 1T PSS | 6W PSS | 1Y PSS | 3Y PSS |
|  | Tau  p | Tau  p | Tau  p | Tau  p | Tau  p | Tau  p | Tau  p | Tau  p |
| 1T Core DS-R | 0.014 0.799 | 0.131 0.061 | -0.066 0.381 | -0.003 0.952 | 0.107 0.087 | **0.173** **0.015** | -0.040 0.601 | 0.038 0.483 |
| 1T Contamination DS-R | 0.041 0.450 | **0.158** **0.024** | -0.042 0.581 | 0.034 0.529 | **0.153** **0.014** | 0.137 0.052 | 0.068 0.365 | 0.019 0.730 |
| 1T Animal Reminder DS-R | 0.091 0.091 | 0.008 0.912 | 0.031 0.684 | 0.052 0.328 | **0.146** **0.020** | **0.149** **0.035** | 0.096 0.205 | 0.070 0.199 |
| 1T Sexual TDDS | -0.001 0.985 | -0.014 0.839 | -0.001 0.990 | -0.010 0.856 | 0.092 0.137 | 0.046 0.505 | 0.051 0.492 | 0.092 0.086 |
| 1T Moral TDDS | -0.035 0.514 | 0.086 0.213 | -0.095 0.200 | 0.007 0.898 | 0.095 0.123 | 0.130 0.062 | -0.092 0.214 | 0.027 0.611 |
| 6W Core DS-R | 0.052 0.450 | 0.023 0.738 | -0.071 0.366 | 0.073 0.285 | **0.186** **0.008** | 0.120 0.083 | 0.030 0.702 | 0.045 0.520 |
| 6W Contamination DS-R | 0.008 0.902 | -0.04 0.555 | -0.125 0.111 | 0.046 0.501 | 0.071 0.313 | -0.023 0.744 | -0.051 0.516 | -0.028 0.693 |
| 6W Animal Reminder DS-R | 0.017 0.800 | -0.033 0.634 | 0.065 0.409 | 0.013 0.854 | **0.239 0.001** | 0.098 0.159 | **0.196** **0.013** | 0.098 0.161 |
| 6W Sexual TDDS | 0.034 0.619 | **0.165 0.017** | -0.044 0.576 | 0.075 0.278 | **0.209 0.003** | **0.173** **0.012** | 0.060 0.453 | 0.038 0.586 |
| 6W Moral TDDS | 0.079 0.251 | -0.088 0.201 | 0.041 0.603 | 0.073 0.290 | 0.046 0.516 | 0.012 0.864 | 0.032 0.684 | **0.151 0.032** |
| 1Y Core DS-R | 0.039 0.599 | -0.04 0.609 | -0.004 0.954 | 0.045 0.546 | 0.136 0.069 | 0.052 0.509 | -0.004 0.959 | 0.014 0.858 |
| 1Y Contamination DS-R | -0.046 0.531 | **-0.174** **0.027** | -0.108 0.144 | -0.016 0.826 | 0.016 0.826 | -0.117 0.142 | 0.001 0.994 | -0.032 0.667 |
| 1Y Animal Reminder DS-R | -0.013 0.861 | -0.052 0.506 | -0.027 0.712 | -0.004 0.961 | **0.256** **0.001** | 0.097 0.221 | 0.130 0.079 | 0.089 0.234 |
| 1Y Sexual TDDS | -0.016 0.832 | 0.046 0.558 | -0.082 0.267 | 0.013 0.859 | 0.070 0.348 | 0.014 0.857 | -0.044 0.551 | 0.005 0.947 |
| 1Y Moral TDDS | -0.066 0.372 | 0.070 0.377 | -0.034 0.644 | -0.012 0.875 | 0.06 0.421 | 0.084 0.289 | -0.052 0.481 | 0.058 0.437 |
| 3Y Core DS-R | 0.059 0.264 | 0.049 0.471 | -0.095 0.197 | 0.098 0.062 | **0.158** **0.010** | 0.085 0.218 | -0.024 0.744 | 0.064 0.232 |
| 3Y Contamination DS-R | 0.041 0.438 | 0.038 0.575 | -0.123 0.096 | 0.042 0.429 | 0.083 0.178 | 0.05 0.469 | -0.094 0.204 | 0.037 0.488 |
| 3Y Animal Reminder DS-R | 0.076 0.151 | 0.024 0.725 | -0.003 0.970 | 0.081 0.127 | **0.192** **0.002** | 0.081 0.240 | 0.066 0.368 | 0.064 0.232 |
| 3Y Sexual TDDS | 0.039 0.464 | 0.077 0.269 | -0.102 0.172 | **0.112** **0.034** | 0.101 0.102 | 0.066 0.347 | -0.083 0.264 | 0.021 0.692 |
| 3Y Moral TDDS | -0.010 0.848 | -0.029 0.681 | -0.053 0.480 | -0.040 0.450 | 0.033 0.591 | 0.035 0.614 | 0.008 0.917 | 0.025 0.647 |

The abbreviations refer to various stages of pregnancy and postpartum recovery: 1T – first trimester of pregnancy, 6W – six weeks postpartum, 1Y – one year postpartum, and 3Y – three years postpartum. Significant correlations are bolded. Those correlations which remained significant after applying Benjamini-Hochberg correction for multiple testing are underlined, FDR was set at 0.1

Table S7: Correlation analysis of maternal disgust and maternal positive and negative affectivity

| Variables | Affectivity | | | | | | | |
| --- | --- | --- | --- | --- | --- | --- | --- | --- |
|  | 1T Positive | 6W Positive | 1Y Positive | 3Y Positive | 1T Negative | 6W Negative | 1Y Negative | 3Y Negative |
|  | Tau  p | Tau  p | Tau  p | Tau  p | Tau  p | Tau  p | Tau  P | Tau  p |
| 1T Core DS-R | 0.036 0.565 | -0.074 0.294 | 0.038 0.613 | 0.009 0.869 | 0.079 0.207 | 0.069 0.329 | -0.055 0.467 | 0.050 0.360 |
| 1T Contamination DS-R | 0.012 0.845 | -0.138 0.051 | -0.134 0.076 | 0.012 0.828 | 0.036 0.567 | 0.112 0.114 | -0.064 0.401 | -0.022 0.685 |
| 1T Animal Reminder DS-R | -0.046 0.462 | **-0.143 0.044** | -0.102 0.179 | -0.072 0.184 | 0.055 0.379 | 0.062 0.382 | 0.011 0.888 | 0.093 0.088 |
| 1T Sexual TDDS | 0.112 0.071 | 0.016 0.824 | 0.008 0.914 | -0.053 0.330 | -0.002 0.969 | 0.046 0.509 | -0.031 0.674 | 0.048 0.375 |
| 1T Moral TDDS | -0.009 0.885 | -0.061 0.383 | -0.009 0.904 | -0.033 0.536 | 0.012 0.852 | 0.108 0.122 | -0.07 0.346 | 0.068 0.207 |
| 6W Core DS-R | -0.066 0.349 | -0.057 0.407 | -0.070 0.373 | -0.026 0.711 | **0.171** **0.015** | 0.015 0.826 | -0.009 0.905 | 0.051 0.465 |
| 6W Contamination DS-R | -0.009 0.901 | -0.028 0.684 | -0.04 0.610 | 0.011 0.874 | -0.023 0.746 | -0.065 0.349 | **-0.167 0.034** | -0.085 0.228 |
| 6W Animal Reminder DS-R | -0.114 0.103 | -0.093 0.179 | **-0.167** **0.034** | -0.117 0.095 | 0.133 0.057 | 0.068 0.326 | 0.129 0.100 | 0.054 0.444 |
| 6W Sexual TDDS | 0.034 0.626 | -0.066 0.343 | -0.096 0.226 | 0.048 0.497 | 0.095 0.177 | **0.137 0.048** | -0.036 0.652 | 0.037 0.603 |
| 6W Moral TDDS | 0.066 0.351 | 0.039 0.569 | 0.011 0.892 | 0.015 0.834 | -0.08 0.256 | 0.013 0.856 | -0.017 0.831 | 0.041 0.559 |
| 1Y Core DS-R | -0.066 0.380 | -0.026 0.748 | -0.066 0.371 | -0.147 0.051 | 0.147 0.050 | -0.055 0.491 | -0.047 0.525 | -0.03 0.687 |
| 1Y Contamination DS-R | -0.038 0.612 | -0.013 0.866 | -0.094 0.202 | -0.069 0.357 | -0.038 0.611 | **-0.239** **0.003** | -0.115 0.121 | -0.109 0.145 |
| 1Y Animal Reminder DS-R | -0.021 0.777 | -0.097 0.221 | -0.123 0.094 | -0.09 0.232 | **0.206 0.006** | 0.044 0.578 | 0.057 0.438 | 0.061 0.414 |
| 1Y Sexual TDDS | 0.045 0.545 | -0.007 0.934 | -0.059 0.421 | 0.044 0.562 | 0.037 0.623 | -0.016 0.840 | -0.137 0.064 | -0.075 0.316 |
| 1Y Moral TDDS | 0.065 0.387 | 0.027 0.731 | **0.165 0.025** | 0.018 0.809 | -0.034 0.653 | 0.072 0.362 | -0.131 0.076 | -0.036 0.631 |
| 3Y Core DS-R | -0.029 0.644 | -0.081 0.241 | -0.092 0.214 | -0.025 0.644 | **0.140 0.023** | 0.021 0.760 | -0.091 0.217 | 0.104 0.054 |
| 3Y Contamination DS-R | 0.028 0.649 | -0.071 0.305 | -0.037 0.612 | 0.002 0.968 | 0.004 0.950 | -0.034 0.628 | -0.123 0.095 | 0.003 0.952 |
| 3Y Animal Reminder DS-R | -0.081 0.189 | -0.135 0.051 | -0.099 0.181 | -0.083 0.124 | **0.131 0.034** | 0.080 0.248 | 0.017 0.814 | 0.092 0.088 |
| 3Y Sexual TDDS | 0.079 0.204 | -0.039 0.575 | 0.045 0.550 | 0.042 0.433 | 0.048 0.442 | 0.035 0.618 | **-0.192 0.010** | 0.028 0.607 |
| 3Y Moral TDDS | 0.048 0.443 | 0.060 0.398 | 0.140 0.062 | -0.011 0.831 | -0.018 0.771 | 0.015 0.832 | **-0.150 0.046** | -0.025 0.646 |

The abbreviations refer to various stages of pregnancy and postpartum recovery: 1T – first trimester of pregnancy, 6W – six weeks postpartum, 1Y – one year postpartum, and 3Y – three years postpartum. Significant correlations are bolded. Those correlations which remained significant after applying Benjamini-Hochberg correction for multiple testing are underlined, FDR was set at 0.1.

Table S8: Correlations between maternal affective states and child disgust

| Variables | Total CDS | | Affect CDS | | Avoidance CDS | |
| --- | --- | --- | --- | --- | --- | --- |
|  | Tau | p | Tau | p | Tau | P |
| 1T STAI | 0.040 | 0.465 | 0.065 | 0.236 | 0.024 | 0.663 |
| 6W STAI | 0.056 | 0.430 | 0.082 | 0.244 | 0.047 | 0.506 |
| 1Y STAI | 0.036 | 0.633 | 0.050 | 0.507 | 0.037 | 0.623 |
| 3Y STAI | 0.008 | 0.878 | 0.040 | 0.462 | 0.005 | 0.933 |
| 1T PSS | 0.085 | 0.176 | 0.089 | 0.157 | 0.065 | 0.301 |
| 6W PSS | 0.107 | 0.139 | **0.147** | **0.040** | 0.074 | 0.302 |
| 1Y PSS | 0.095 | 0.208 | 0.037 | 0.629 | 0.058 | 0.442 |
| 3Y PSS | 0.088 | 0.114 | 0.042 | 0.448 | 0.087 | 0.113 |
| 1T PA | -0.032 | 0.612 | -0.053 | 0.400 | -0.023 | 0.715 |
| 6W PA | -0.137 | 0.058 | -0.098 | 0.171 | **-0.143** | **0.046** |
| 1Y PA | **-0.150** | **0.048** | **-0.173** | **0.022** | -0.090 | 0.233 |
| 3Y PA | -0.025 | 0.649 | -0.021 | 0.707 | -0.025 | 0.653 |
| 1T NA | -0.040 | 0.529 | 0.013 | 0.836 | -0.050 | 0.424 |
| 6W NA | 0.040 | 0.583 | 0.079 | 0.272 | 0.014 | 0.849 |
| 1Y NA | -0.027 | 0.724 | -0.026 | 0.736 | -0.056 | 0.458 |
| 3Y NA | -0.002 | 0.973 | -0.004 | 0.937 | 0.000 | 0.997 |

The abbreviations refer to various stages of pregnancy and postpartum: 1T – first trimester of pregnancy, 6W – six weeks postpartum, 1Y – one year postpartum, and 3Y – three years postpartum. Significant correlations are bolded. No correlation remained significant after applying Benjamini-Hochberg correction for multiple testing, FDR was set at 0.1.
